# Interhospital critical care transfer delays result from organisational not geographical factors: secondary analysis of deteriorating ward patients in 49 UK hospitals

**DOI:** 10.1186/cc14590

**Published:** 2015-03-16

**Authors:** DJ Wong, SK Harris

**Affiliations:** 1 King's College Hospital, London, UK; 2 University College London, UK

## Introduction

Critically ill patients may require interhospital transfer for specialist care or because of a lack of local ICU capacity. Harm is assumed from delays that result, but it is not clear whether these delays are due to transfer distances or deficiencies in the organisation of care.

## Methods

**Figure 1:**
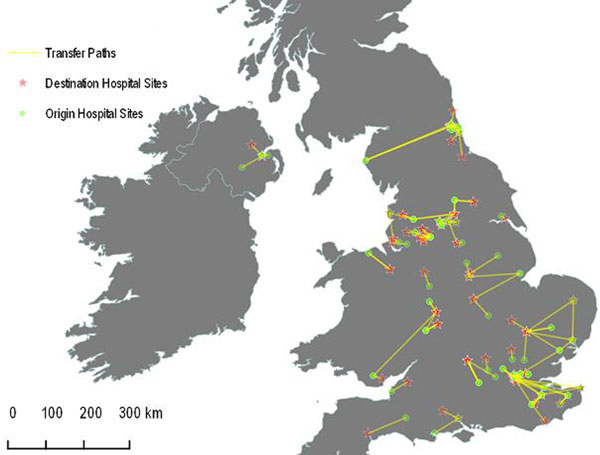
**Map of transfers**.

In total, 151 of 15,602 deteriorating ward patients in the (SPOT)light study [1] were transferred rather than admitted locally. We defined delay as the time from critical care assessment in the first hospital to arrival in critical care in the second hospital. We used hospital postcodes to derive latitude and longitude, and calculated both geodesic (straight-line) distances (Figure 1) and road distances between the sites using R version 3.1.1 [2]. We compared daytime versus overnight (7:00 pm to 7:00 am) transfer durations assuming traffic would contribute less to delay overnight. Mapping and visualisation was performed on Quantum GIS version 2.4 [3].

## Results

**Figure 2:**
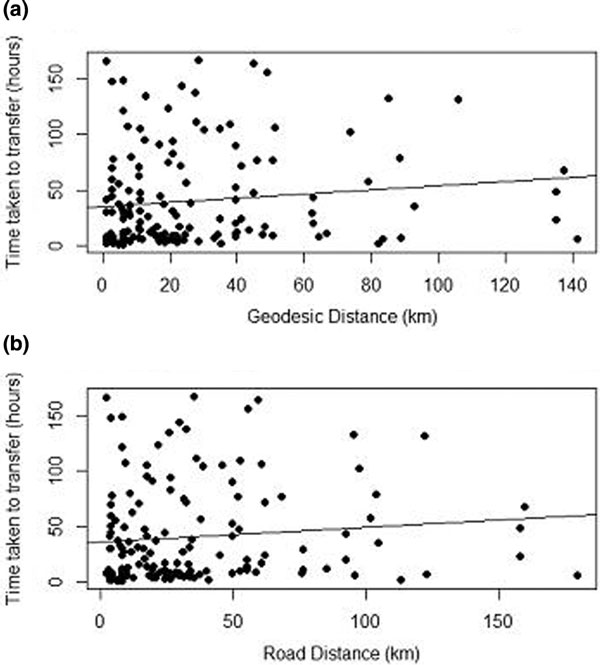
****(a) **Time duration versus geodesic distance (*R*^2^ = 0**.015). **(b) **Time duration versus road distance (*R*^2^ = 0.011).

The median delay to admission was 22 hours (range 41 to 167 hours). The median geodesic distance was 18 km (range 1 to 141 km), and road distance was 24 km (range 2 to 180 km). Correlations between time delay and geodesic/road distances were weak (Figure 2, *R*^2 ^= 0.015 and 0.011, respectively). Transfer delays in the daytime and overnight were similar (Wilcoxon rank sum, *P *= 0.6).

## Conclusion

Interhospital transfers are subject to clinically significant delays, and substantial travel distances. Delays are only weakly correlated to distances travelled and may reflect delays resulting from organisational inefficiencies. We infer that efforts to improve the efficiency of transfer should focus on local organisational issues. There was no difference in the duration taken for overnight versus daytime transfers.
